# Profiles of 71 Human Milk Oligosaccharides and Novel Sub-Clusters of Type I Milk: Results from the Ulm SPATZ Health Study

**DOI:** 10.3390/nu17020280

**Published:** 2025-01-14

**Authors:** Zhuoxin Peng, Linda P. Siziba, Marko Mank, Bernd Stahl, John Gonsalves, Deborah Wernecke, Dietrich Rothenbacher, Jon Genuneit

**Affiliations:** 1 Pediatric Epidemiology, Department of Pediatrics, Medical Faculty, Leipzig University, Liebigstr 20a, Haus 6, 04103 Leipzig, Germany; linda.siziba@medizin.uni-leipzig.de (L.P.S.); jon.genuneit@medizin.uni-leipzig.de (J.G.); 2 Danone Research & Innovation, 3584 CT Utrecht, The Netherlands; marko.mank@danone.com (M.M.); bernd.stahl@danone.com (B.S.); john.gonsalves@danone.com (J.G.); 3 Department of Chemical Biology & Drug Discovery, Faculty of Science, Utrecht Institute for Pharmaceutical Sciences, Utrecht University, 3584 CG Utrecht, The Netherlands; 4 Institute of Epidemiology and Medical Biometry, Ulm University, 89075 Ulm, Germany; deborah.wernecke@uni-ulm.de (D.W.); dietrich.rothenbacher@uni-ulm.de (D.R.); 5 German Center for Child and Adolescent Health (DZKJ), 89075 Ulm, Germany; 6 German Center for Child and Adolescent Health (DZKJ), 04103 Leipzig, Germany

**Keywords:** human milk oligosaccharides, lactation, liquid chromatography, mass spectrometry, concentration, milk type

## Abstract

Background/Objectives: Although approximately 160 human milk oligosaccharides (HMOs) have been identified, current studies on HMO quantitation are limited to the 10–19 most abundant HMOs. We assessed the variations in the relative concentrations of 71 HMO structures over lactation in human milk samples by an advanced liquid chromatography–mass spectrometry approach. Methods: Samples were collected from 64 mothers at 6 weeks, 6 months, and 12 months of lactation in the Ulm SPATZ Health Study, a German birth cohort. In this longitudinal study, we fitted linear mixed-effect models to analyze changes in the log2-transformed and standardized HMO concentration over time. Based on the profile of 71 HMOs, we also fitted a group-based multi-trajectory (GBMT) model to cluster mothers secreting cluster type I milk, who account for the majority of lactating mothers. Results: We found that 52 HMOs had a decreasing trend (regression coefficients ranging from −1.41 to −0.17) and 9 had an increasing trend (regression coefficients ranging from 0.25 to 0.64) during lactation, and the findings were statistically significant after multiple testing corrections. Using human milk samples of 49 mothers with type I milk, we further identified two novel sub-clusters with distinct longitudinal trajectories of concentrations of 71 HMOs during lactation: Type I-a (N = 20) and I-b (N = 29). These sub-clusters were not associated with maternal non-genetic characteristics. Conclusions: Our findings extend existing knowledge about the structural diversity of HMOs and their variations over lactation. These may pave the way to investigate the potential nutritional benefits of various HMOs on infant health and early life development in the future.

## 1. Introduction

Human milk oligosaccharides (HMOs) are a family of glycans that account for the third most abundant fraction of biomolecules in human milk, after lactose and fat [1]. HMOs convey several potential nutritional benefits to breastfed infants, including early life modulation of the immune system, shaping of the gut microbiome, infant growth, and brain development [2,3,4,5]. The velocity of infant growth is known to be positively correlated with the concentration of fucosylated and sialylated HMOs in human milk [6]. A higher level of 2′-fucosyllactose (2′-FL) and lacto-N-difucohexaose was reported to have a protective effect on infant diarrhea [7]. The level of 2′-FL in human milk at 1 month was also found to be associated with better infant cognitive development at the age of 2 years [8].

HMO concentrations are influenced by maternal genetic and non-genetic factors, especially by the expression of secretor and Lewis genes [9]. The two most important determinants for individual HMO levels are the maternal secretor gene (Se gene, FUT2) and the Lewis gene (Le gene, FUT3) [10]. The active secretor gene translates into syntheses of α1-2-fucosyltransferase, which determines levels of α1,2-fucosylated HMOs, like 2′-FL or lacto-N-fucopentaose I (LNFP-I). The Lewis gene influences the secretion of α1,3-and α1,4-fucosylated HMOs, e.g., lacto-N-fucopentaose II (LNFP II) [10]. Based on the status of Se and Le genes, and subsequently the related presence of specific HMOs, human milk from lactating mothers is commonly categorized into four milk groups or milk types: Type I (Se+Le+), II (Se-Le+), III (Se+Le-), and IV (Se-Le-) [9,11]. Type I milks contain the broadest structural diversity of HMOs [9], whereas other milk types may either present very minute amounts of certain HMOs or completely lack them, as Se and/or Le genes may not be active [10]. Previous studies suggested that type I milk is the most predominant milk group amongst lactating mothers [12,13]. The lactation stage is the most frequently reported non-genetic factor influencing the concentration of HMOs [14,15]. The concentrations of total and most individual HMOs reportedly decrease over the lactation period. Other maternal characteristics that potentially influence HMO concentrations include maternal body mass index (BMI), maternal age, maternal diet, parity, mode of delivery, and gestational age [16].

Until now, more than 160 HMOs have been identified in human milk [17,18]. However, the diversity of HMO structures remains underestimated [19]. Current studies on the distribution of HMOs and/or their association with infant health outcomes are mostly based on the quantification of the 10–19 most abundant HMOs [17,20,21,22,23]. To the best of our knowledge, only one study has investigated up to 24 HMOs [24]. Evidence for quantitative changes of other more complex HMOs over the course of lactation is sparse. To fill this gap, we quantified relative concentrations of 71 HMOs in 6-week, 6-month, and 12-month human milk samples from the Ulm SPATZ Health Study in Germany, using advanced liquid chromatography and electrospray ionization quadrupole time-of-flight mass spectrometry (LC-ESI-QTOF-MS) [25].

Our study aim was to assess the trajectories of those 71 HMOs over time of lactation based on their relative concentrations. This is the novelty of our study, as many of these more complex HMOs have been never characterized in this context before. Furthermore, there are only four well-established and known human milk types that can be distinguished. We therefore also aimed to explore novel unobserved HMO sub-clusters and the maternal determinants based on the extended investigation of 71 HMOs. The quantification of more HMO isomers might provide more opportunities to identify associations of HMOs with infants’ health outcomes.

## 2. Materials and Methods

### 2.1. Cohort Overview

The Ulm SPATZ Health Study recruited 970 mothers and their 1006 newborn infants shortly after delivery at the University Medical Center Ulm (which was the only maternity hospital at that time in Ulm, Germany) between April 2012 and May 2013. Demographic, lifestyle, and health information were collected at baseline using self-administered questionnaires and from pregnancy-related healthcare documentation. Additional study-related information after delivery was collected using questionnaires sent via post at 6 weeks, 6 months, and 1 year (overall response rate was 49%). Mothers who were <18 years old, did not have sufficient German language mastery/skill, had childbirth in outpatient settings, or had a stillbirth were excluded. Mothers were also excluded if they or their infants were sent to intensive care units after delivery. More details about the cohort have been published elsewhere [26]. Ethical approval for SPATZ was obtained from the Ethics Board of Ulm University (No. 311/11).

### 2.2. Human Milk Samples and HMO Quantification

Trained nurses visited participants’ homes and collected human milk samples (foremilk) that were secreted by mothers on the same day or the previous day at the 6-week, 6-month, and 12-month follow-ups. The process of breastmilk sample collection has been described in a previous publication [23]. All human milk samples were stored at −80 °C until analysis. The current study is restricted to a total of 192 human milk samples that were available from 64 mothers at all three time points (6-week, 6-month, and 12-month). Human milk samples were transferred to the Danone Research & Innovation laboratory on dry ice and analyzed. The human milk samples were processed prior to the LC-ESI-QTOF-MS analysis as follows. To 100 µL of defrosted human milk sample, 300 µL of 1.3% formic acid was added and vortexed. Acidification of HM samples by formic acid was carried out to improve protein separation in the subsequent centrifugation step. The samples were refrigerated at 4–8 °C for 60 min and centrifuged for 15 min. Then, 75 µL of the intermediate layer was diluted 10 times with water and further cleaned by MCX SPE cartridge. The cleaned extracts were diluted 1:2 with acetonitrile, centrifuged, and injected into an LC-ESI-IM-QTOF-MS system. The system consisted of a Waters I-Class UPLC coupled to a Waters Vion High-Resolution Mass Spectrometer (Waters, Milford, MA, USA). Chromatographic separation of the HMOs was achieved on two Waters Premier Glycan Columns (Waters, Milford, MA, USA) at 67.5 C. Eluents A and B consisted, respectively, of 20% acetonitrile and 80% acetonitrile, both containing 50 mM ammonium formate with an approximated pH of 4.4. Measurements were carried out in negative ion sensitivity mode using the all-ion fragmentation (AIF) approach, operating with a mass resolution of >25,000, to identify and quantify individual HMOs in addition to a retention time-based separation and identification of compounds. A total HMO fraction which was isolated from pooled human milk and lactose depleted was used for the relative quantitation of a total of 73 HMOs. A 10 mg/mL solution of this total HMO fraction was serially diluted and used to construct calibration functions for 73 HMOs. The estimated sensitivity or limit of detection of our LC-ESI-QTOF-MS approach for HMOs is at least >1 microgram/mL, based on known concentrations of low abundant HMOs like 3′-galactosyllactose (3′-GL), which are fully detectable by our approach. A minimum of 6 calibration points in the calibration function were selected per individual HMO. For the calibration function, weighing 1/x and quadratic fit per compound were applied.

The four commonly known human milk types were assigned to individual milk samples based on the quantification of the following HMOs: (i) milk with relative concentrations of LNFP I and lacto-N-difucohexaose I (LNDFH I) below the lower limit of quantification (LLOQ) was categorized as Type II, (ii) milk with relative concentrations of LNFP II and LNDFH I below LLOQ was categorized as Type III, (iii) milk with relative concentrations of LNFP I, LNFP II, and LNDFH I below LLOQ was categorized as Type IV, and (iv) all other milk was categorized as Type I.

### 2.3. Statistical Analysis

Among 73 quantified HMOs, two HMOs were excluded from the statistical analysis: b-pentasaccharide had relative concentrations <LLOQ for more than 90% of the samples, and lacto-N-difucohexaose II had relative concentrations above the upper limit of quantification (>ULOQ) in more than 30% of the samples. For the remaining 71 HMOs, data < LLOQ were regarded as missing or imputed as LLOQ/√2 if appropriate (see below for details); data > ULOQ were imputed based on the extrapolation of the HMO-specific calibration line.

We used linear mixed-effect models to analyze the change in the relative concentration of each HMO (outcome variable) over time (independent variable). Each HMO concentration at one time point was log2-transformed and scaled to a normalized value with a mean value of 0 and a standard deviation (SD) of 1. The mixed-effect model included time as a continuous variable, maternal baseline characteristics that might influence the components of human milk, batches, and a random intercept for each mother. Data on maternal characteristics from questionnaires included maternal age at baseline, maternal BMI before pregnancy, parity (1 vs. >1), gestational age, education levels (high school or lower vs. university degree or higher), smoking before pregnancy (yes vs no), and delivery mode (vaginal delivery vs. cesarean delivery). In the fixed-effect models for the change of HMOs over time, all data < LLOQ were regarded as missing values so that the estimates of HMOs would not be influenced by those mothers who cannot secrete them. We used a false discovery rate (FDR) of 0.01 as the α-threshold for statistical significance to correct for multiple comparisons. The HMOs that changed over time on a level of statistical significance were ranked and presented according to the coefficient for time.

To identify clusters beyond known milk types, we fitted a group-based multi-trajectory (GBMT) model to cluster type I milk, which accounts for the majority of lactating mothers, based on the trajectories of the 71 HMOs over time. The GBMT model assesses multiple-dimensional indicators (i.e., the 71 relative HMO concentrations) integrally over time and it identifies clusters of observations (i.e., mothers) that have a similar pattern in those multiple-dimensional indicators [27]. We fitted the GBMT model with the R package gbmt [28]. The relative concentrations of 71 HMOs were log2-transformed, scaled to set the mean as 0 and SD as 1, and adjusted for batch effect by quantile normalization per each time point with the R package batchtma prior to fitting to GBMT models. For the clustering model, data < LLOQ were imputed as LLOQ/√2. Since we only had three time points, we arbitrarily set the polynomial degree of the trajectory function as 1 (linear trajectory) and used the Bayesian information criterion (BIC) as the criteria to identify the optimal number of clusters. We further judged the appropriateness of the GBMT model by average posterior probability (ideally above 0.7) and predicted probability of group membership (ideally above 5%). Based on the new HMO sub-clusters among mothers with type I milk above, we explored the potential maternal factors that are associated with the sub-clusters and tested the associations by ANOVA, chi-squared test, or Fisher exact test if appropriate.

Finally, since most of the analyzed 71 HMOs are not commercially available in sufficient purity and quantity, we evaluated whether the profile of 12 of the most abundant and commercially available HMOs at the first two time points can be used as proxies to predict these sub-clusters based on all 71 HMOs at three time points. The absolute concentrations of 2′-FL, 3-fucosyllactose (3-FL), 3′-sialyllactose (3′-SL), 6′-galactosyllactose (6′-GL), 2′,3-difucosyllactose (DFL), 6′-sialyllactose (6-′SL), lacto-N-tetraose (LNT), LNFP-I, LNFP-II, lacto-N-fucopentaose III (LNFP-III), lacto-N-fucopentaose V (LNFP-V), and LNDFH I at 6 weeks and 6 months, measured in our previous study, were used to develop the prediction model [23]. We randomly split the 49 mothers with type I milk into equal halves as a training set and a validation set. We constructed logistic regressions to fit the data in the training set with four strategies: (1) direct use of absolute concentrations of 12 HMOs at 6 weeks and 6 months (24 predictors); (2) use of log2-transformed and scaled absolute concentration of 12 HMOs at 6 weeks and 6 months (24 predictors); (3) use of concentration differences of individual HMOs between 6 weeks and 6 months (12 predictors); and (4) restriction to the part of 12 HMOs that changed statistically significantly during lactation according to the mixed-effect models as predictors. We replicated the prediction models in the validation set to test the performance of the predictions. We constructed receiver operating characteristic (ROC) curves and estimated the area under the receiver operating curve (AUC) and 95% confidence intervals (CIs) after 2000 bootstrap resampling. *p* values shown are all two-sided. Statistical analyses were performed using SAS version 9.4 (The SAS Institute, Cary, NC, USA) and R (version 4.3.1; R Foundation for Statistical Computing).

## 3. Results

### 3.1. Basic Characteristics

**Figure 1:**
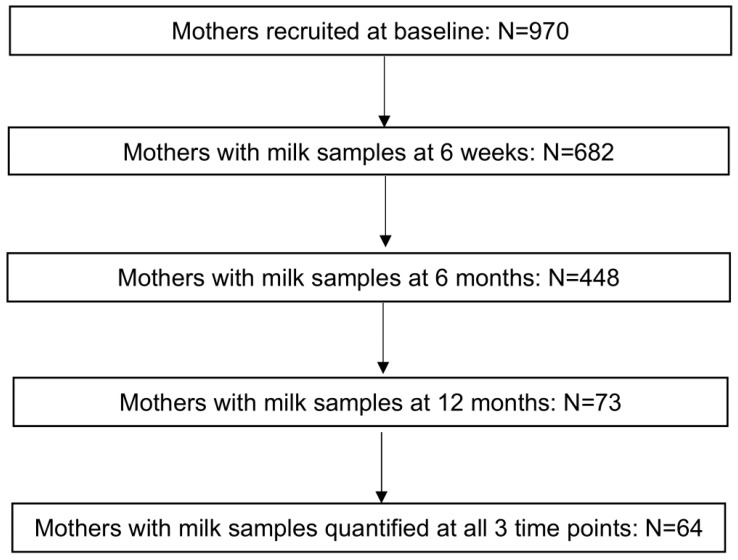
Flowchart of the inclusion of mothers from the baseline cohort.

**Figure 2:**
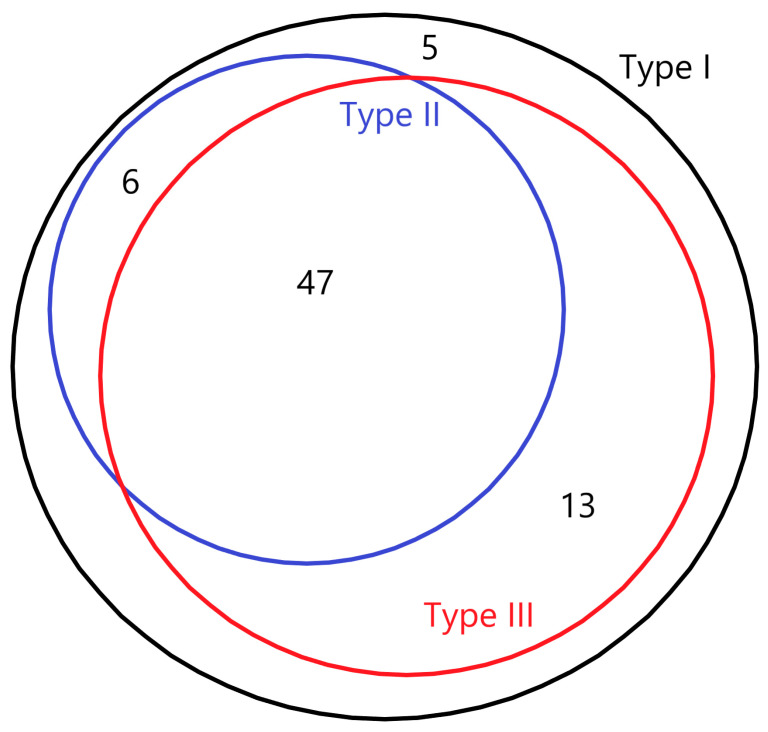
The overlap of the secretion of 71 HMOs in type I, II, and III milk.

**Figure 3:**
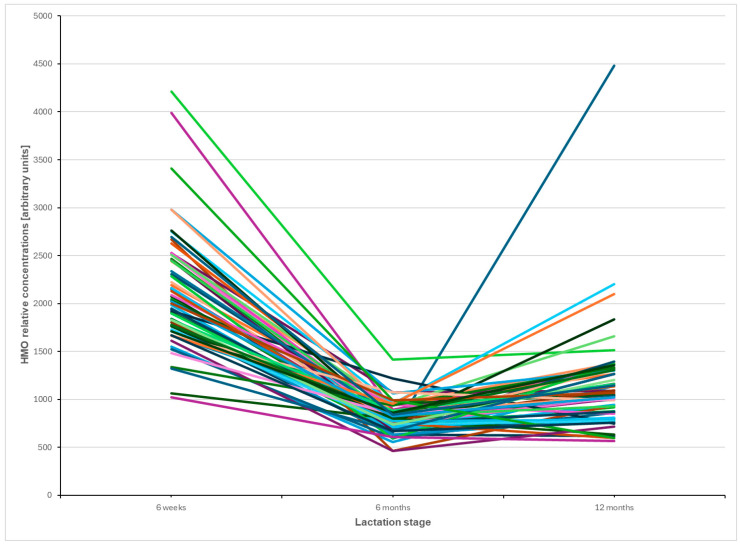
Trajectories of total HMO relative concentrations per lactating mothers over three time points. Abbreviations: HMO: human milk oligosaccharide. Each line represents the HMO concentrations of a mother.

The flowchart of the inclusion of mothers from the baseline cohort is shown in Figure 1. Table 1 depicts and compares the basic characteristics between mothers recruited in the baseline cohort and the lactating mothers included in our study. Among the 64 mothers in this study, 49 (76.6%) had type I milk, 10 (15.6%) had type II milk, 5 (7.8%) had type III milk, and no mothers had type IV milk. Supplementary Table S1 shows the mean of relative concentrations of 71 HMOs among milk types I through III, with their degrees of polymerization (DP) ranging from 2 to 11 over time. All 71 HMOs were identified in type I milk; 53 HMOs were identified in type II milk; 60 HMOs were identified in type III milk; and 5 of those HMOs were not identified in either type II or type III milk (i.e., lacto-N-difucohexaose I, trifucosyl-lacto-N-tetraose X5, trifucosyllacto-N-hexaose I, difucosylsialyl-lacto-N-hexaose X2, and a-heptasaccharide). The overlap of the secretion of 71 HMOs among type I, II, and III milk is shown in a Venn diagram (Figure 2). Figure 3 displays the trajectories of total HMO concentration change per lactating mother over time. A line in Figure 3 represents the change in HMO concentration of each mother at three time points.

### 3.2. Trajectories of Individual HMOs over Time

**Figure 4:**
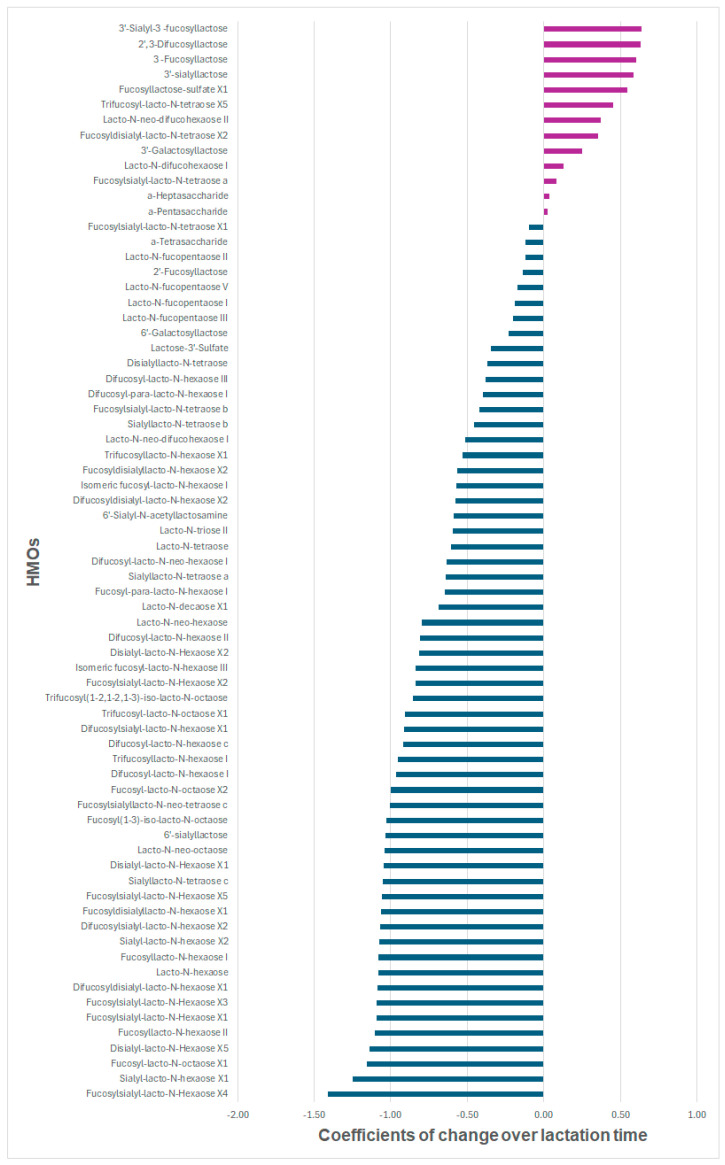
Estimated regression coefficients for change of relative concentrations over time among 71 HMOs. The regression coefficients of time points are based on mixed-effect models with a random intercept for each mother that accounted for maternal age at baseline, maternal BMI before pregnancy, parity (1 vs. >1), gestational age (weeks), mothers’ education level (high school or lower vs. university degree or higher), maternal smoking before pregnancy (yes vs. no), delivery mode (vaginal delivery vs. cesarean delivery), and batch effect. Abbreviations: HMOs: human milk oligosaccharides.

We assessed the changes in the 71 HMOs over three time points (6 weeks, 6 months, and 12 months) during lactation. Figure 4 illustrates the estimated regression coefficients of changes in relative concentrations over lactation time among 71 HMOs in the mixed-effect models. The mixed-effect models show that the relative concentrations of 9 HMOs (3′-sialyl-3-fucosyllactose, DFL, 3-FL, 3′-SL, fucosyl-lacto-N-sulfate X1, trifucosyl-lacto-N-tetraose X5, lacto-N-neo-difucohexaose II, fucosyldisialyl-lacto-N-tetraose X2, and 3′-galactosyllactose) had a statistically significant increasing trend during lactation, and 52 had a statistically significant decreasing trend during lactation, after correction for multiple testing (FDR < 0.01) and adjustment for potential confounders (Supplementary Table S2). Table 2 illustrates all the HMOs (N = 9) with a significantly increasing trend and the top 10 HMOs with a significantly decreasing trend, ranked by effect size. We also performed sensitivity analyses on the changes in HMO concentration between 6 weeks and 6 months (Supplementary Table S3) and between 6 months and 12 months (Supplementary Table S4).

### 3.3. HMO Clustering

**Figure 5:**
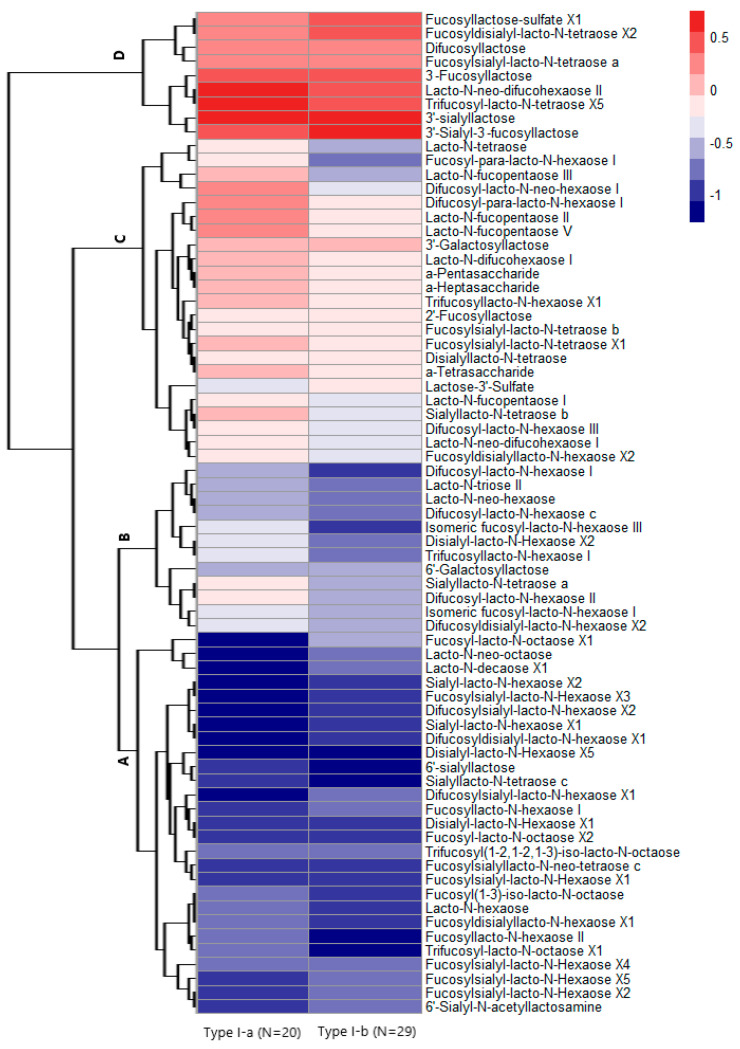
Sub-clustering of 49 lactating mothers with type I milk based on group-based multi-trajectory models. Colors in the cells represent the values of the slope of fitted mixed-effect linear trajectory for the 71 HMOs’ concentrations over time. The regression coefficients of time points are based on mixed-effect models with a random intercept for each mother. Type I-a and type I-b represent two sub-clusters of 49 lactating mothers with milk type I. The letters A–D highlighted in the clustering tree represent four groups of HMOs. Abbreviations: HMOs: human milk oligosaccharides.

We chose a 2-cluster solution for GBMT models, as this solution had the best model meeting the selection criteria. The results of further clustering 49 mothers with type I milk based on GBMT are shown in Figure 5. The color of each cell in the heat map indicates the effect size of the linear trajectory over time for the relative concentrations of the 71 HMOs fitted by mixed-effect models. We identified two potential underlying sub-clusters of type I milk: Type I-a (N = 20) and Type I-b (N = 29). According to distinct patterns of trajectories among two sub-clusters of mothers with type I milk, the 71 HMOs could also be divided into 4 groups (Group A-D in Figure 5) by hierarchical clustering.

Overall, Group A and B have a decreasing trend over lactation whereas both Group C and D have an increasing trend. Group A HMOs (e.g., 6′-SL) are characterized by a similar steep decrease during lactation among both mothers of sub-cluster type I-a and type I-b. Group B HMOs (e.g., trifucosyllacto-N-hexaose I) are characterized by a moderate decrease among cluster type I-a mothers but a steep decrease in cluster type I-b mothers. Group C HMOs (e.g., LNFP-V) are characterized by a steep increase among cluster type I-a mothers but a moderate increase in cluster type I-b mothers. Group D HMOs (e.g., 3′-SL) are characterized by a similar steep increase during lactation among both mothers of cluster type I-a and type I-b. We tested the differences in the maternal characteristics across two sub-clusters of type I milk and did not find any statistically significant differences (Table 3).

**Figure 6:**
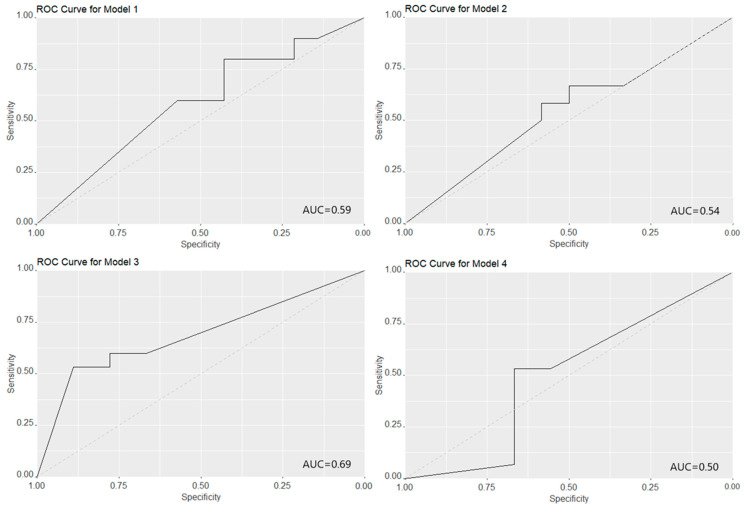
The receiver operating characteristic (ROC) curve and area under the ROC curve (AUC) for predicting the two sub-clusters of mothers secreting type I milk using 12 abundant human milk oligosaccharide (HMO) absolute concentrations at 6 weeks and 6 months in the validation set. Model 1: directly using all absolute concentrations of 12 HMOs at 6 weeks and 6 months (24 predictors); Model 2: using the log2-transformed and scaled absolute concentration of 12 HMOs at 6 weeks and 6 months (24 predictors); Model 3: using the concentration differences of individual HMOs between 6 weeks and 6 months (12 predictors); Model 4: restricted to 8 HMOs that changed statistically significantly during lactation (16 predictors).

Among the 12 most abundant HMOs, eight HMOs (3-FL, 3′-SL, 6′-GL, DFL, 6′-SL, LNT, LNFP-I, and LNFP-V) changed significantly over the lactation period and are therefore included as predictors in the prediction models for the sub-clusters (modelling strategy 4). When we tried to use the 12 most abundant HMOs to predict the sub-clusters, we found that all four prediction strategies had a perfect prediction performance for sub-clusters (AUC = 1) in the training set. However, all four prediction strategies had poor prediction performance in the validation set (See Figure 6). The model strategies using raw absolute HMO concentrations at the first two time points (24 predictors), log2-transformed absolute HMO concentrations at the first two time points (24 predictors), 12 differences of absolute HMO concentrations over the first two time points (12 predictors), and raw absolute concentrations of eight HMOs that changed significantly over time (16 predictors), resulted in AUCs of 0.59 (95% CIs: 0.36–0.81), 0.54 (95% CIs: 0.32–0.77), 0.69 (95% CIs: 0.49–0.89), and 0.50 (95% CIs: 0.24–0.75), respectively.

## 4. Discussion

Using longitudinal data from 64 lactating mothers in the Ulm SPATZ Health Study, we assessed the change of relative concentrations of 71 HMOs over three time points during lactation and identified novel sub-clusters of type I milk. We found that most of these 71 HMOs had a decreasing relative concentration over the 6-week, 6-month, and 12-month time points. Using human milk samples of 49 mothers with type I milk, which represented the majority of analyzed milk samples in this study, we identified two sub-clusters (types I-a and I-b) with distinct HMO structure-dependent longitudinal trajectories of relative HMO concentrations over lactation. The HMOs within these sub-clusters could also be categorized into four groups (groups A–D). Compared with mothers with type I-a milk, mothers with type I-b milk had a steeper decreasing trend in HMO group B and a more moderate increasing trend in HMO group C. We did not find any statistically significant differences in the characteristics of mothers across the type I-a and I-b sub-clusters. Using only the absolute concentration of the 12 most abundant HMOs at 6 weeks and 6 months could not satisfactorily predict the two new sub-clusters based on 71 HMOs.

We previously investigated the change of absolute concentrations of the 16 most abundant HMOs in a larger sample size (n = 682) and found that the concentrations of most HMOs decreased over lactation; only some specific HMOs, like 3-FL, DFL, and 3′-SL, had increasing trends, similar to the present study [23]. Other studies in different regions also observed a similar pattern of HMO changes during lactation [29,30,31,32]. The mechanisms of the increase or decrease of certain HMO concentrations are complex and still not fully understood. A potential explanation is that different HMO structures are synthesized by different glycosyltransferases. The decreasing or increasing activities of those enzymes during a lactation period will lead to the decrease or increase of certain HMO structures [14]. Some studies suggested that HMO changes varied among maternal phenotypes [32,33]. In the present study, we confirmed the relationship of the lactation stage with an extended list of 71 HMOs based on a more advanced LC-ESI-IM-QTOF-MS HMO quantification technique. To our knowledge, our study describes the largest number of HMO structures quantified over different stages of lactation to date. So far, we are only aware of one other study that investigated up to 24 HMOs [24]. In our study, we could identify multiple isomers of certain HMOs that have never been reported nor measured in other studies. For example, only one difucosyl-lacto-N-hexaose (DFLNH) isomer was commonly analyzed in previous studies on HMO compositions [12,34,35], while we included six DFLNH isomers (DFLNH I, difucosyl-lacto-N-neo-hexaose I, difucosyl-para-lacto-N-hexaose I, difucosyl-lacto-N-hexaose II, difucosyl-lacto-N-hexaose III, and difucosyl-lacto-N-hexaose c) in our study. Whether these newly identified HMO isomers are associated with infant health outcomes remains to be studied in the future. Additional research is needed to better understand the correlations and interactions among the changes as well as the functions of these HMOs over lactation.

Furthermore, we made full use of the quantification of 71 relative HMO concentrations and identified two novel sub-clusters of type I milk with distinct HMO profiles that have never been identified before. Mothers with type I-b milk had a steeper decreasing trend in HMO group B and a more moderate increasing trend in HMO group C than mothers with type I-a milk. These findings imply that HMO profiles of future dietary supplements for infants could be better tailored to certain HMO compositions which we found at different stages of lactation. It is interesting to see that those two sub-clusters are not significantly associated with maternal demographic, perinatal, or lifestyle factors. This result may suggest that these maternal characteristics may not be the key determinants of the underlying sub-clusters or that the limited number in our study resulted in insufficient power to show an association. A cohort study in Europe with a larger sample size (N = 290) on 20 major HMOs concluded that the concentration of individual HMOs significantly differed by maternal BMI, delivery mode, and parity, but the differences were quite small [15]. We hypothesize that the sub-clustering of type I milk could be mainly due to genetic factors that were not assessed in the current study. Further studies with larger sample sizes and more comprehensive maternal lifestyle and environmental factors are needed to verify our hypothesis. Our findings also require confirmatory studies based on different regions, as McGuire et al. indicated that the profiles of HMOs produced by mothers varied geographically [12]. Besides, whether the infants of mothers who do not produce specific HMO structures are at higher risk of infections or other implications for early life health remains to be studied.

A reason why previous researchers only focused on a limited number of HMOs is that the 10–15 most abundant HMOs account for 70–75% of total HMO concentrations, and they seem to be sufficient representatives for the pattern of overall HMO profiles [17,36]. However, our study challenges this notion. We found that using the 12 most abundant HMOs had a poor performance (AUC < 0.7) for predicting the sub-clusters of mothers secreting type I milk, based on 71 HMOs. The results revealed the need for extended investigation of more HMO structures, as the whole picture of the HMO profile might be much more complex than the 10–19 most abundant HMO proxies present.

There are several limitations, but also some strengths, of our study. First, we had a small sample of mothers, but with 12-month longitudinal data from their human milk samples to investigate trajectories based on three time points during lactation. Second, the low prevalence of type II, III, and IV milk precluded further analysis of sub-clusters of mothers with these milk types. Due to the extremely low prevalence of type IV milk (1% in the population) [9], we did not identify any type IV milk samples within our cohort. Third, statistically non-significant differences in maternal characteristics in the type I-a and I-b HMO sub-clusters could have been due to insufficient statistical power. The poor performance (AUC values < 0.7) of the prediction models for sub-clusters might be also attributed to the limited sample size and statistical power. Moreover, we lacked maternal diet information and, thus, could not investigate its influence on these sub-clusters of type I milk. Nonetheless, these sub-clusters remain novel and provide more information on relative HMO concentrations. Most of the 71 HMOs are not commercially available, and we lack the pure standards of these HMOs for absolute quantification; this hinders a direct comparison of absolute concentrations across different HMOs. The lack of commercial availability of some HMOs also means that, if future studies find that some newly identified HMOs are beneficial for infant growth, the industry will first have to find ways to produce them to be able to add them to formula, which might be a challenge for the transition from academia to industry. Despite that, we are reporting the largest number of HMOs ever relatively quantified. With the advanced LC-ESI-IM-QTOF-MS technique, we have identified multiple isomers that have not been reported previously. The imputation of data < LLOQ and the extrapolation of data > ULOQ may result in inaccurate relative concentration estimates. The estimated concentrations of HMOs are calculated from HMO-specific calibration functions. The calibration functions may not fit well for data < LLOQ and data > ULOQ, and the bias may vary among different HMOs. Furthermore, we regarded data < LLOQ as the result of the non-secretion of certain HMOs in the linear mixed-effect models. However, data < LLOQ might also be due to the secretion of very low amounts of individual HMOs by mothers and therefore may introduce misclassification bias for milk type.

## 5. Conclusions

In conclusion, we identified and quantified 71 HMOs using advanced liquid chromatography and electrospray ionization ion mobility quadrupole time-of-flight mass spectrometry. Although most of these partly complex HMOs decreased over lactation, nine HMOs showed increasing trends over lactation. We further identified novel HMO-based sub-clusters of type I milk. These two sub-clusters showed distinct trajectories of HMO profiles and were not associated with investigated maternal (non-genetic) factors. Using the absolute concentration of the 12 most abundant HMOs poorly predicted the sub-clusters. These results suggest the need to analyze the accessible pattern of HMOs up to a degree of polymerization of eleven (DP = 11) in future studies. Our findings extend existing knowledge about the structural diversity of HMOs and their profiles present in human milk. They might also pave the way to investigate the potential nutritional benefits of various HMOs, including complex structures up to DP = 11, on infant health and early life development in the future.

## Figures and Tables

**Table 1:** Characteristics of lactating mothers who provided three human milk samples in the Ulm SPATZ Health Study (n = 64) vs. study baseline (n = 970).

Maternal Characteristics	n (%) or Means ± SDs	
	In this Analysis	Baseline Cohort
Total	64 (100)	970 (100)
Milk Types		
I	49 (76.6)	-
II	10 (15.6)	-
III	5 (7.88)	-
IV	0 (0)	-
Age at baseline, years	34.3 ± 4.0	32.7 ± 4.8
Education level		
University degree or higher	49 (77.8)	561 (59.0)
High school or lower	14 (22.2)	390 (41.0)
Smoking before pregnancy		
Yes	7 (11.1)	259 (27.0)
No	56 (88.9)	700 (73.0)
BMI before pregnancy	24.2 ± 4.4	24.8 ± 5.1
Gestational age, weeks	39.2 ± 1.4	38.8 ± 1.7
Parity		
1	27 (42.2)	522 (53.9)
>1	37 (57.8)	447 (46.1)
Delivery mode		
Vaginal (spontaneous or assisted)	50 (78.1)	709 (73.2)
Cesarean (emergency or elective)	14 (21.9)	260 (26.8)

Abbreviations: SD: standard deviation; BMI: body mass index.

**Table 2:** Human milk oligosaccharide (HMO) with statistically significant increasing/decreasing trends over lactation, ranked by the coefficient values of time. N = 192 observations from 64 subjects.

HMOs	Coef. ^a^	95% CIs	P_FDR_ ^b^
**All 9 HMOs with increasing trends**			
3′-Sialyl-3-fucosyllactose	0.64	0.52; 0.75	2.35E-19
2′,3-Difucosyllactose	0.63	0.50; 0.77	4.73E-15
3-Fucosyllactose	0.60	0.52; 0.68	7.55E-29
3′-Sialyllactose	0.59	0.47; 0.71	5.86E-17
Fucosyllactose-sulfate X1	0.54	0.37; 0.72	2.75E-08
Trifucosyl-lacto-N-tetraose X5	0.45	0.27; 0.64	9.43E-06
Lacto-N-neo-difucohexaose II	0.37	0.26; 0.49	8.41E-09
Fucosyldisialyl-lacto-N-tetraose X2	0.35	0.23; 0.48	3.30E-07
3′-Galactosyllactose	0.25	0.07; 0.43	9.95E-03
**Top 10 HMOs with decreasing trends**			
Fucosylsialyl-lacto-N-Hexaose X4	−1.41	−1.93; −0.90	1.70E-04
Sialyl-lacto-N-hexaose X1	−1.25	−1.63; −0.87	2.43E-06
Fucosyl-lacto-N-octaose X1	−1.16	−1.51; −0.81	2.80E-05
Disialyl-lacto-N-Hexaose X5	−1.14	−1.22; −1.05	2.96E-43
Fucosyllacto-N-hexaose II	−1.10	−1.21; −0.99	2.77E-39
Fucosylsialyl-lacto-N-Hexaose X1	−1.09	−1.20; −0.99	3.72E-36
Fucosylsialyl-lacto-N-Hexaose X3	−1.09	−1.28; −0.91	6.87E-14
Difucosyldisialyl-lacto-N-hexaose X1	−1.09	−1.21; −0.97	8.28E-22
Lacto-N-hexaose	−1.08	−1.23; −0.93	2.45E-23
Fucosyllacto-N-hexaose I	−1.08	−1.28; −0.88	9.16E-13

^a^. Coef. refers to the coefficients of time point term in the mixed-effect models with a random intercept for each mother that accounted for maternal age at baseline, maternal BMI before pregnancy, parity (1 vs. >1), gestational age (weeks), mothers’ education level (high school or lower vs. university degree or higher), maternal smoking before pregnancy (yes vs. no), delivery mode (vaginal delivery vs. cesarean delivery), and batch effect. ^b^. P_FDR_ refers to false discovery rate adjusted *p* values. P_FDR_ < 0.01 is considered as the statistical significance threshold. Abbreviations: HMOs: human milk oligosaccharides; Coef.: coefficient; CIs: confidence intervals; FDR: false discovery rate.

**Table 3:** Maternal characteristics at baseline of two sub-clusters of type I milk (N = 49).

Maternal Characteristics	No. (%) or Means ± SDs		Test Statistics	Degrees of Freedom	*p*
	Type I-a (n = 20)	Type I-b (n = 29)			
Maternal age at baseline, years	34.1 ± 2.9	34.7 ± 3.9	0.33	1	0.57
Have a university degree	15 (75.0)	22 (78.6)	0.084	1	0.77
Smoking before pregnancy: yes	3 (15.0)	4 (14.3)	0.0048	1	1.00
Maternal BMI before pregnancy	24.5 ± 4.6	23.3 ± 3.8	0.87	1	0.35
Gestational age, week	38.9 ± 1.5	39.5 ± 1.2	2.44	1	0.13
Parity > 1	10 (50.0)	18 (62.1)	0.70	1	0.40
Cesarean	6 (30.0)	5 (17.2)	1.11	1	0.32

Abbreviations: SDs: standard deviation, BMI: body mass index. Test statistics are F-values for continuous characteristics or chis-square values for categorical characteristics.

## Data Availability

The study was based on cohort data from the Ulm SPATZ Health Study (https://www.ulmer-forschen.de/, accessed on 11 January 2025), which could be accessed through application to and approval from J.G (Jon Genuneit). and D.R. The lab data are available on reasonable request from the corresponding author.

## Supplementary Materials

The following supporting information can be downloaded at: https://www.mdpi.com/article/10.3390/nu17020280/s1, Table S1. Means of 71 HMO relative concentrations and the degrees of polymerization (DP) in Type I, II, and III lactating mothers over time. Table S2. Detailed results regarding the effect of time points on the relative concentrations of 71 HMOs during three lactation stages. Table S3. Detailed results regarding the effect of time points on the relative concentrations of 71 HMOs between 6 weeks and 6 months. Table S4. Detailed results regarding the effect of time points on the relative concentrations of 71 HMOs between 6 months and 12 months.
