# Supplementary material for: Profiles of 71 Human Milk Oligosaccharides and Novel Sub-Clusters of Type I Milk: Results from the Ulm SPATZ Health Study
Source: Nutrients. 2025 Jan 14;17(2):280. doi: 10.3390/nu17020280 (PMC11767774; doi:10.3390/nu17020280)
Supplement: Supplementary file 1 [file nutrients-17-00280-s001.zip › nutrients-3375200-supplementary.pdf]

Supplementary materials

Table S1. Means* of 71 HMO relative concentrations and the degrees of polymerization (DP) in Type I, II, and III lactating mothers over time.

HMOs	DP	Type I	Type II	Type III
2'-Fucosyllactose	3	37.5	-	56.2
3-Fucosyllactose	3	50.2	96.1	15.8
3'-sialyllactose	3	18.8	16.5	14.2
6'-sialyllactose	3	19.2	19.6	25.6
Lactose-3'-Sulfate	2	10.6	11.3	9.7
6'-Galactosyllactose	3	15.5	18.5	16.0
3'-Galactosyllactose	3	3.5	4.0	4.5
2',3-Difucosyllactose	4	29.5	-	11.8
3'-Sialyl-3-fucosyllactose	4	40.9	124.2	7.1
Fucosyllactose-sulfate X1	3	40.6	-	36.3
Lacto-N-tetraose	4	14.7	16.8	12.1
Lacto-N-fucopentaose I	5	15.7	-	30.9
Lacto-N-fucopentaose II	5	16.3	42.5	-
Lacto-N-fucopentaose III	5	20.2	20.7	19.4
Lacto-N-fucopentaose V	5	13.6	49.3	3.8
Sialyllacto-N-tetraose a	5	15.1	11.3	14.6
Sialyllacto-N-tetraose b	5	17.3	28.0	11.9
Sialyllacto-N-tetraose c	5	20.5	21.1	18.6
Lacto-N-difucohexaose I	6	27.3	-	-
Lacto-N-neo-difucohexaose I	6	13.9	-	16.3
Lacto-N-neo-difucohexaose II	6	22.3	72.1	6.2
Disialyllacto-N-tetraose	6	28.1	35.3	15.4
Fucosylsialyl-lacto-N-tetraose a	6	30.1	74.0	2.3
Fucosylsialyl-lacto-N-tetraose b	6	18.2	4.2	17.0
Fucosylsialyllacto-N-neo-tetraose c	6	20.2	57.0	5.5
Fucosylsialyl-lacto-N-tetraose X1	6	18.0	33.4	9.1
Trifucosyl-lacto-N-tetraose X5	7	30.0	-	-
Fucosyldisialyl-lacto-N-tetraose X2	7	35.1	215.3	4.8
Lacto-N-hexaose	6	23.4	21.5	25.0
Lacto-N-neo-hexaose	6	18.8	7.1	15.9
Difucosyl-lacto-N-hexaose I	8	13.0	-	28.2
Fucosyl-para-lacto-N-hexaose I	7	11.8	9.6	8.7
Fucosyllacto-N-hexaose II	7	17.7	24.6	13.9
Isomeric fucosyl-lacto-N-hexaose I	7	12.0	-	15.2
Fucosyllacto-N-hexaose I	7	17.3	-	18.8
Isomeric fucosyl-lacto-N-hexaose III	7	13.9	10.0	8.1
Sialyl-lacto-N-hexaose X1	7	66.1	111.3	46.4

Sialyl-lacto-N-hexaose X2	7	35.7	18.9	26.6
Difucosyl-lacto-N-neo-hexaose I	8	17.3	19.6	14.9
Difucosyl-para-lacto-N-hexaose I	8	12.1	36.1	-
Difucosyl-lacto-N-hexaose II	8	14.8	43.2	-
Difucosyl-lacto-N-hexaose III	8	8.5	-	20.4
Difucosyl-lacto-N-hexaose c	8	14.9	3.0	0.9
Fucosylsialyl-lacto-N-Hexaose X1	8	23.7	38.8	16.2
Fucosylsialyl-lacto-N-Hexaose X2	8	22.3	24.2	25.2
Fucosylsialyl-lacto-N-Hexaose X3	8	22.3	1.7	40.9
Fucosylsialyl-lacto-N-Hexaose X4	8	41.0	189.7	-
Fucosylsialyl-lacto-N-Hexaose X5	8	31.6	40.6	16.5
Disialyl-lacto-N-Hexaose X1	8	39.4	15.1	27.6
Disialyl-lacto-N-Hexaose X2	8	27.1	13.7	16.4
Disialyl-lacto-N-Hexaose X5	8	30.0	35.9	30.3
Trifucosyllacto-N-hexaose I	9	19.0	-	-
Trifucosyllacto-N-hexaose X1	9	15.0	5.3	-
Difucosylsialyl-lacto-N-hexaose X1	9	20.1	-	24.8
Difucosylsialyl-lacto-N-hexaose X2	9	48.8	-	-
Fucosyldisialyllacto-N-hexaose X1	9	24.5	26.8	19.4
Fucosyldisialyllacto-N-hexaose X2	9	23.5	24.4	16.0
Difucosyldisialyl-lacto-N-hexaose X1	10	37.3	6.8	34.7
Difucosyldisialyl-lacto-N-hexaose X2	10	31.1	91.5	-
Lacto-N-neo-octaose	8	17.8	11.3	11.0
Fucosyl(1-3)-iso-lacto-N-octaose	9	12.0	15.8	6.9
Fucosyl-lacto-N-octaose X1	9	16.2	-	19.9
Fucosyl-lacto-N-octaose X2	9	16.8	16.1	9.6
Trifucosyl(1-2,1-2,1-3)-iso-lacto-N-octaose	11	9.4	1.5	21.4
Trifucosyl-lacto-N-octaose X1	11	16.4	8.6	4.8
Lacto-N-decaose X1	10	25.8	25.3	17.7
Lacto-N-triose II	3	4.8	5.4	3.9
6'-Sialyl-N-acetyllactosamine	3	21.2	24.2	22.4
a-Tetrasaccharide	4	16.2	-	35.8
a-Pentasaccharide	5	30.6	-	11.1
a-Heptasaccharide	7	27.8	-	-

Abbreviations: HMOs: Human milk oligosaccharides
* If the mothers with type II and III did not secrete a specific HMO, the cell would be “-”.

Table S2. Detailed results regarding the effect of time points on the relative concentrations of 71 HMOs during three lactation stages

**HMOs**	**Coef.** [a]	**95% CIs**	**Raw P values**	P_{FDR} [b]
Fucosylsialyl-lacto-N-Hexaose X4	-1.41	-1.93; -0.90	1.34E-04	1.70E-04
Sialyl-lacto-N-hexaose X1	-1.25	-1.63; -0.87	1.71E-06	2.43E-06
Fucosyl-lacto-N-octaose X1	-1.16	-1.51; -0.81	2.17E-05	2.80E-05
Disialyl-lacto-N-Hexaose X5	-1.14	-1.22; -1.05	1.25E-44	2.96E-43
Fucosyllacto-N-hexaose II	-1.10	-1.21; -0.99	1.95E-40	2.77E-39
Fucosylsialyl-lacto-N-Hexaose X1	-1.09	-1.20; -0.99	3.14E-37	3.72E-36
Fucosylsialyl-lacto-N-Hexaose X3	-1.09	-1.28; -0.91	2.71E-14	6.87E-14
Difucosyldisialyl-lacto-N-hexaose X1	-1.09	-1.21; -0.97	2.10E-22	8.28E-22
Lacto-N-hexaose	-1.08	-1.23; -0.93	5.53E-24	2.45E-23
Fucosyllacto-N-hexaose I	-1.08	-1.28; -0.88	4.00E-13	9.16E-13
Sialyl-lacto-N-hexaose X2	-1.07	-1.29; -0.86	1.32E-11	2.76E-11
Difucosylsialyl-lacto-N-hexaose X2	-1.07	-1.19; -0.95	3.86E-18	1.19E-17
Fucosyldisialyllacto-N-hexaose X1	-1.06	-1.14; -0.98	1.63E-57	5.79E-56
Fucosylsialyl-lacto-N-Hexaose X5	-1.06	-1.37; -0.74	5.98E-08	9.87E-08
Sialyllacto-N-tetraose c	-1.05	-1.14; -0.96	2.42E-43	4.30E-42
Disialyl-lacto-N-Hexaose X1	-1.05	-1.15; -0.94	2.61E-32	2.06E-31
Lacto-N-neo-octaose	-1.04	-1.25; -0.83	1.32E-10	2.47E-10
6'-sialyllactose	-1.03	-1.11; -0.96	8.06E-58	5.72E-56
Fucosyl(1-3)-iso-lacto-N-octaose	-1.03	-1.15; -0.91	6.38E-33	5.66E-32
Fucosylsialyllacto-N-neo-tetraose c	-1.01	-1.12; -0.90	2.78E-36	2.82E-35

Fucosyl-lacto-N-octaose X2	-1.00	-1.27; -0.72	3.21E-09	5.84E-09
Difucosyl-lacto-N-hexaose I	-0.97	-1.07; -0.86	3.77E-30	2.68E-29
Trifucosyllacto-N-hexaose I	-0.95	-1.08; -0.82	4.88E-27	2.89E-26
Difucosyl-lacto-N-hexaose c	-0.92	-1.04; -0.80	2.06E-26	1.13E-25
Difucosylsialyl-lacto-N-hexaose X1	-0.91	-1.06; -0.76	1.18E-19	3.99E-19
Trifucosyl-lacto-N-octaose X1	-0.90	-1.02; -0.79	3.13E-26	1.59E-25
Trifucosyl(1-2,1-2,1-3)-iso-lacto-N-octaose	-0.85	-0.99; -0.72	1.21E-18	3.91E-18
Fucosylsialyl-lacto-N-Hexaose X2	-0.84	-1.12; -0.55	1.05E-05	1.38E-05
Isomeric fucosyl-lacto-N-hexaose III	-0.84	-0.99; -0.69	3.27E-20	1.22E-19
Disialyl-lacto-N-Hexaose X2	-0.82	-0.95; -0.69	5.96E-24	2.49E-23
Difucosyl-lacto-N-hexaose II	-0.81	-0.94; -0.69	1.34E-24	6.34E-24
Lacto-N-neo-hexaose	-0.80	-0.95; -0.64	1.89E-16	5.37E-16
Lacto-N-decaose X1	-0.69	-1.21; -0.17	2.70E-02	3.04E-02
Fucosyl-para-lacto-N-hexaose I	-0.65	-0.81; -0.48	1.52E-12	3.37E-12
Sialyllacto-N-tetraose a	-0.64	-0.78; -0.50	5.57E-16	1.52E-15
Difucosyl-lacto-N-neo-hexaose I	-0.64	-0.79; -0.48	3.32E-13	7.86E-13
Lacto-N-tetraose	-0.61	-0.77; -0.44	3.82E-11	7.33E-11
Lacto-N-triose II	-0.59	-0.75; -0.43	1.61E-11	3.27E-11
6'-Sialyl-N-acetyllactosamine	-0.59	-0.74; -0.44	3.19E-12	6.86E-12
Difucosyldisialyl-lacto-N-hexaose X2	-0.58	-0.72; -0.43	2.56E-11	5.05E-11
Isomeric fucosyl-lacto-N-hexaose I	-0.57	-0.76; -0.38	4.69E-08	7.93E-08
Fucosyldisialyllacto-N-hexaose X2	-0.56	-0.70; -0.43	6.38E-14	1.56E-13
Trifucosyllacto-N-hexaose X1	-0.53	-0.72; -0.35	1.21E-07	1.91E-07
Lacto-N-neo-difucohexaose I	-0.51	-0.69; -0.33	1.33E-07	2.05E-07

Sialyllacto-N-tetraose b	-0.45	-0.61; -0.30	7.93E-08	1.28E-07
Fucosylsialyl-lacto-N-tetraose b	-0.42	-0.57; -0.27	1.48E-07	2.24E-07
Difucosyl-para-lacto-N-hexaose I	-0.40	-0.56; -0.24	2.81E-06	3.91E-06
Difucosyl-lacto-N-hexaose III	-0.38	-0.53; -0.23	3.50E-06	4.78E-06
Disialyllacto-N-tetraose	-0.37	-0.51; -0.23	5.00E-07	7.24E-07
Lactose-3'-Sulfate	-0.35	-0.52; -0.17	1.55E-04	1.93E-04
6'-Galactosyllactose	-0.23	-0.37; -0.10	1.04E-03	1.27E-03
Lacto-N-fucopentaose III	-0.20	-0.37; -0.03	2.15E-02	2.46E-02
Lacto-N-fucopentaose I	-0.19	-0.33; -0.06	6.12E-03	7.36E-03
Lacto-N-fucopentaose V	-0.17	-0.30; -0.05	6.96E-03	8.24E-03
2'-Fucosyllactose	-0.14	-0.27; -0.01	4.34E-02	4.74E-02
Lacto-N-fucopentaose II	-0.12	-0.26; 0.02	8.33E-02	8.96E-02
a-Tetrasaccharide	-0.12	-0.23; -0.01	3.90E-02	4.32E-02
Fucosylsialyl-lacto-N-tetraose X1	-0.10	-0.23; 0.03	1.35E-01	1.43E-01
a-Pentasaccharide	0.02	-0.10; 0.14	6.98E-01	6.98E-01
a-Heptasaccharide	0.04	-0.11; 0.18	6.02E-01	6.11E-01
Fucosylsialyl-lacto-N-tetraose a	0.08	-0.04; 0.20	1.79E-01	1.87E-01
Lacto-N-difucohexaose I	0.13	-0.06; 0.31	1.90E-01	1.96E-01
3'-Galactosyllactose	0.25	0.07; 0.43	8.55E-03	9.95E-03
Fucosyldisialyl-lacto-N-tetraose X2	0.35	0.23; 0.48	2.23E-07	3.30E-07
Lacto-N-neo-difucohexaose II	0.37	0.26; 0.49	4.74E-09	8.41E-09
Trifucosyl-lacto-N-tetraose X5	0.45	0.27; 0.64	7.04E-06	9.43E-06
Fucosyllactose-sulfate X1	0.54	0.37; 0.72	1.59E-08	2.75E-08
3'-sialyllactose	0.59	0.47; 0.71	1.98E-17	5.86E-17

3-Fucosyllactose	0.60	0.52; 0.68	1.17E-29	7.55E-29
2',3-Difucosyllactose	0.63	0.50; 0.77	1.80E-15	4.73E-15
3'-Sialyl-3-fucosyllactose	0.64	0.52; 0.75	6.62E-20	2.35E-19

a. Coef. refers to the coefficients of time point term in the mixed-effect models with a random intercept for each mother that accounted for maternal age during pregnancy, maternal BMI before pregnancy, parity (1 vs >1), mothers' gestational age (weeks), mothers' education level (high school or lower vs. university degree or higher), maternal smoking before pregnancy (yes vs no), delivery mode (vaginal delivery vs. cesarean delivery), and batch effect.

b. P_{FDR} refers to false discovery rate adjusted P values. P_{FDR} <0.01 is considered as the statistical significance threshold

Abbreviations: HMOs: Human milk oligosaccharides; Coef. : coefficient; CIs: confidence intervals; FDR: false discovery rate

Table S3. Detailed results regarding the effect of time points on the relative concentrations of 71 HMOs between 6 weeks and 6 months

HMOs	Coef. [a]	95% CIs	Raw P values	P_{FDR} [b]
Fucosylsialyl-lacto-N-Hexaose X2	-2.25	-2.72; -1.79	8.04E-07	1.27E-06
Sialyl-lacto-N-hexaose X1	-2.20	-2.48; -1.91	1.80E-16	6.73E-16
Difucosylsialyl-lacto-N-hexaose X2	-2.12	-2.30; -1.93	9.68E-20	5.29E-19
Fucosylsialyl-lacto-N-Hexaose X4	-2.08	-2.82; -1.33	4.45E-05	6.20E-05
Fucosyldisialyllacto-N-hexaose X1	-2.02	-2.16; -1.87	8.68E-44	6.16E-42
Fucosylsialyl-lacto-N-Hexaose X1	-1.88	-2.10; -1.65	8.16E-20	4.83E-19
Fucosylsialyl-lacto-N-Hexaose X3	-1.87	-2.14; -1.60	9.73E-12	2.30E-11
Difucosylsialyl-lacto-N-hexaose X1	-1.84	-2.12; -1.56	1.68E-17	7.95E-17
Fucosyldisialyllacto-N-hexaose X2	-1.84	-2.12; -1.55	2.64E-22	1.87E-21
Lacto-N-decaose X1	-1.81	-2.78; -0.85	3.72E-02	4.26E-02
Fucosylsialyl-lacto-N-Hexaose X5	-1.80	-2.31; -1.30	8.83E-09	1.69E-08
Disialyl-lacto-N-Hexaose X5	-1.75	-1.89; -1.60	1.90E-37	6.75E-36
Sialyl-lacto-N-hexaose X2	-1.73	-1.99; -1.46	1.71E-13	4.86E-13
Difucosyl-lacto-N-hexaose I	-1.72	-1.95; -1.48	2.66E-18	1.35E-17
Trifucosyllacto-N-hexaose I	-1.69	-1.94; -1.44	3.79E-20	2.45E-19
Fucosyllacto-N-hexaose II	-1.67	-1.85; -1.48	1.16E-30	1.65E-29
Difucosyl-lacto-N-hexaose c	-1.65	-1.93; -1.36	4.62E-17	1.93E-16
Difucosyldisialyl-lacto-N-hexaose X1	-1.60	-1.79; -1.42	6.54E-06	9.88E-06
Sialyllacto-N-tetraose c	-1.59	-1.74; -1.43	7.19E-34	1.70E-32

Fucosyl(1-3)-iso-lacto-N-octaose	-1.57	-1.77; -1.37	4.79E-24	3.78E-23
Trifucosyl-lacto-N-octaose X1	-1.56	-1.79; -1.32	2.43E-17	1.08E-16
Fucosylsialyllacto-N-neo-tetraose c	-1.51	-1.70; -1.32	1.06E-25	1.25E-24
Sialyllacto-N-tetraose a	-1.50	-1.72; -1.29	4.21E-24	3.74E-23
Disialyl-lacto-N-Hexaose X1	-1.48	-1.72; -1.25	8.79E-17	3.47E-16
6'-Sialyl-N-acetyllactosamine	-1.47	-1.67; -1.28	1.50E-24	1.52E-23
Lactose-3'-Sulfate	-1.45	-1.73; -1.18	2.89E-15	1.03E-14
Lacto-N-neo-octaose	-1.43	-1.82; -1.04	3.76E-07	6.36E-07
Trifucosyllacto-N-hexaose X1	-1.40	-1.76; -1.03	1.41E-10	3.23E-10
Fucosyl-lacto-N-octaose X2	-1.40	-1.84; -0.95	6.18E-07	9.97E-07
Trifucosyl(1-2,1-2,1-3)-iso-lacto-N-octaose	-1.38	-1.76; -1.01	4.65E-07	7.68E-07
Disialyllacto-N-tetraose	-1.35	-1.63; -1.06	6.76E-15	2.29E-14
Fucosyl-lacto-N-octaose X1	-1.31	-2.08; -0.54	1.15E-02	1.34E-02
Difucosyldisialyl-lacto-N-hexaose X2	-1.27	-1.66; -0.89	3.74E-08	6.81E-08
Lacto-N-hexaose	-1.27	-1.53; -1.01	9.73E-14	2.88E-13
Fucosyllacto-N-hexaose I	-1.26	-1.75; -0.77	8.80E-05	1.20E-04
Disialyl-lacto-N-Hexaose X2	-1.26	-1.52; -0.99	3.07E-14	9.48E-14
Fucosylsialyl-lacto-N-tetraose b	-1.24	-1.54; -0.95	6.51E-12	1.59E-11
6'-sialyllactose	-1.21	-1.33; -1.08	6.08E-33	1.08E-31
Difucosyl-lacto-N-hexaose III	-1.19	-1.53; -0.86	4.17E-09	8.22E-09
Fucosyl-para-lacto-N-hexaose I	-1.15	-1.42; -0.88	1.76E-12	4.46E-12
Fucosylsialyl-lacto-N-tetraose X1	-1.15	-1.42; -0.88	9.32E-13	2.45E-12
Sialyllacto-N-tetraose b	-1.11	-1.42; -0.81	2.95E-10	6.22E-10
Difucosyl-lacto-N-hexaose II	-1.11	-1.35; -0.87	1.93E-13	5.27E-13

Difucosyl-lacto-N-neo-hexaose I	-1.07	-1.40; -0.73	1.27E-08	2.37E-08
Lacto-N-triose II	-0.89	-1.25; -0.53	5.02E-06	7.75E-06
Fucosylsialyl-lacto-N-tetraose a	-0.89	-1.12; -0.65	2.98E-10	6.22E-10
Lacto-N-neo-difucohexaose I	-0.84	-1.19; -0.49	1.16E-05	1.68E-05
Lacto-N-neo-hexaose	-0.84	-1.18; -0.50	8.97E-06	1.33E-05
6'-Galactosyllactose	-0.82	-1.04; -0.60	2.52E-10	5.59E-10
Lacto-N-tetraose	-0.79	-1.02; -0.57	5.29E-10	1.07E-09
Isomeric fucosyl-lacto-N-hexaose III	-0.77	-1.05; -0.50	3.07E-07	5.32E-07
Isomeric fucosyl-lacto-N-hexaose I	-0.56	-0.90; -0.22	2.23E-03	2.78E-03
Lacto-N-fucopentaose V	-0.53	-0.76; -0.30	2.31E-05	3.28E-05
Difucosyl-para-lacto-N-hexaose I	-0.51	-0.82; -0.19	2.31E-03	2.83E-03
3'-sialyllactose	-0.50	-0.68; -0.33	1.91E-07	3.39E-07
Lacto-N-fucopentaose I	-0.49	-0.79; -0.19	2.09E-03	2.65E-03
3'-Galactosyllactose	-0.48	-0.82; -0.13	8.38E-03	9.91E-03
Fucosyldisialyl-lacto-N-tetraose X2	-0.37	-0.61; -0.12	4.38E-03	5.27E-03
Lacto-N-fucopentaose II	-0.17	-0.44; 0.09	1.99E-01	2.18E-01
a-Heptasaccharide	-0.16	-0.46; 0.15	3.20E-01	3.45E-01
a-Tetrasaccharide	-0.01	-0.26; 0.24	9.38E-01	9.41E-01
a-Pentasaccharide	-0.01	-0.21; 0.20	9.41E-01	9.41E-01
Lacto-N-difucohexaose I	0.02	-0.36; 0.41	8.99E-01	9.26E-01
2'-Fucosyllactose	0.03	-0.23; 0.29	8.10E-01	8.46E-01
Trifucosyl-lacto-N-tetraose X5	0.08	-0.29; 0.44	6.90E-01	7.32E-01
3'-Sialyl-3-fucosyllactose	0.20	-0.01; 0.40	6.09E-02	6.86E-02
Fucosyllactose-sulfate X1	0.30	-0.04; 0.64	8.41E-02	9.33E-02

Lacto-N-neo-difucohexaose II	0.50	0.21; 0.79	1.26E-03	1.63E-03
2',3-Difucosyllactose	0.50	0.26; 0.75	1.78E-04	2.38E-04
Lacto-N-fucopentaose III	0.54	0.26; 0.83	3.67E-04	4.83E-04
3-Fucosyllactose	0.93	0.75; 1.12	1.07E-14	3.45E-14

a. Coef. refers to the coefficients of time point term in the mixed-effect models with a random intercept for each mother that accounted for maternal age during pregnancy, maternal BMI before pregnancy, parity (1 vs >1), mothers' gestational age (weeks), mothers' education level (high school or lower vs. university degree or higher), maternal smoking before pregnancy (yes vs no), delivery mode (vaginal delivery vs. cesarean delivery), and batch effect.

b. P_{FDR} refers to false discovery rate adjusted P values. P_{FDR} <0.01 is considered as the statistical significance threshold

Abbreviations: HMOs: Human milk oligosaccharides; Coef. : coefficient; CIs: confidence intervals; FDR: false discovery rate

Table S4. Detailed results regarding the effect of time points on the relative concentrations of 71 HMOs between 6 months and 12 months

**HMOs**	**Coef.** [a]	**95% CIs**	**Raw P values**	**P_{FDR}** [b]
Lacto-N-hexaose	-1.09	-1.52; -0.66	6.43E-06	4.57E-05
Fucosyl-lacto-N-octaose X1	-1.03	-1.58; -0.48	1.42E-02	3.05E-02
Fucosylsialyl-lacto-N-Hexaose X5	-1.02	-2.57; 0.53	2.31E-01	3.22E-01
Lacto-N-fucopentaose III	-1.00	-1.46; -0.55	3.22E-05	2.08E-04
Isomeric fucosyl-lacto-N-hexaose III	-0.89	-1.34; -0.43	2.45E-04	1.02E-03
Fucosyllacto-N-hexaose II	-0.85	-1.19; -0.50	6.31E-06	4.57E-05
Difucosyl-lacto-N-hexaose II	-0.74	-1.11; -0.38	1.24E-04	5.86E-04
Fucosylsialyl-lacto-N-Hexaose X1	-0.74	-1.08; -0.40	7.09E-05	4.19E-04
Difucosyl-lacto-N-neo-hexaose I	-0.66	-1.01; -0.31	3.36E-04	1.23E-03
Fucosyllacto-N-hexaose I	-0.60	-1.14; -0.07	3.60E-02	7.07E-02
Fucosylsialyl-lacto-N-Hexaose X3	-0.60	-1.01; -0.20	6.68E-03	1.58E-02
Difucosyl-lacto-N-hexaose I	-0.59	-0.99; -0.18	6.11E-03	1.55E-02
Fucosyl(1-3)-iso-lacto-N-octaose	-0.53	-0.91; -0.16	6.66E-03	1.58E-02
Lacto-N-triose II	-0.53	-0.89; -0.17	4.91E-03	1.40E-02
Disialyl-lacto-N-Hexaose X1	-0.51	-0.82; -0.20	2.17E-03	6.69E-03
Lacto-N-neo-hexaose	-0.48	-0.91; -0.05	3.05E-02	6.18E-02
Sialyl-lacto-N-hexaose X2	-0.48	-0.96; 0.00	6.43E-02	1.17E-01
6'-sialyllactose	-0.47	-0.71; -0.24	1.34E-04	5.94E-04

Disialyl-lacto-N-Hexaose X5	-0.47	-0.69; -0.25	8.73E-05	4.77E-04
Fucosylsialyllacto-N-neo-tetraose c	-0.44	-0.76; -0.12	7.53E-03	1.73E-02
Difucosyldisialyl-lacto-N-hexaose X1	-0.44	-0.81; -0.06	3.68E-02	7.07E-02
Lacto-N-neo-octaose	-0.43	-1.14; 0.28	2.55E-01	3.42E-01
Lacto-N-tetraose	-0.43	-0.90; 0.04	7.82E-02	1.39E-01
Sialyllacto-N-tetraose c	-0.35	-0.63; -0.06	1.83E-02	3.83E-02
Trifucosyl(1-2,1-2,1-3)-iso-lacto-N-octaose	-0.34	-0.82; 0.15	1.78E-01	2.75E-01
Fucosyldisialyllacto-N-hexaose X1	-0.31	-0.55; -0.07	1.36E-02	3.02E-02
Sialyl-lacto-N-hexaose X1	-0.29	-1.20; 0.62	9.35E-01	9.62E-01
Trifucosyllacto-N-hexaose I	-0.29	-0.65; 0.07	1.18E-01	2.04E-01
Difucosyl-para-lacto-N-hexaose I	-0.27	-0.71; 0.17	2.36E-01	3.22E-01
Disialyl-lacto-N-Hexaose X2	-0.24	-0.62; 0.13	2.10E-01	3.11E-01
Difucosyl-lacto-N-hexaose c	-0.21	-0.55; 0.13	2.34E-01	3.22E-01
Trifucosyl-lacto-N-octaose X1	-0.19	-0.49; 0.12	2.29E-01	3.22E-01
Fucosyl-para-lacto-N-hexaose I	-0.18	-0.66; 0.31	4.79E-01	5.67E-01
Difucosylsialyl-lacto-N-hexaose X2	-0.14	-0.49; 0.21	4.39E-01	5.29E-01
Fucosyl-lacto-N-octaose X2	-0.12	-0.96; 0.72	7.88E-01	8.22E-01
Isomeric fucosyl-lacto-N-hexaose I	0.02	-0.57; 0.60	9.55E-01	9.69E-01
Lacto-N-fucopentaose II	0.07	-0.36; 0.49	7.61E-01	8.07E-01
Fucosylsialyl-lacto-N-tetraose b	0.10	-0.34; 0.53	6.55E-01	7.05E-01
Difucosyldisialyl-lacto-N-hexaose X2	0.11	-0.36; 0.58	6.51E-01	7.05E-01
Sialyllacto-N-tetraose b	0.11	-0.32; 0.54	6.11E-01	6.77E-01
Lacto-N-decaose X1	0.13	-5.70; 5.95	1.00E+00	1.00E+00
Lacto-N-fucopentaose I	0.14	-0.32; 0.59	5.56E-01	6.37E-01

Lacto-N-neo-difucohexaose I	0.16	-0.36; 0.69	5.51E-01	6.37E-01
Sialyllacto-N-tetraose a	0.16	-0.22; 0.55	4.11E-01	5.11E-01
Difucosylsialyl-lacto-N-hexaose X1	0.18	-0.20; 0.56	3.63E-01	4.77E-01
a-Tetrasaccharide	0.19	-0.29; 0.68	4.39E-01	5.29E-01
2'-Fucosyllactose	0.20	-0.25; 0.65	3.79E-01	4.89E-01
Trifucosyllacto-N-hexaose X1	0.21	-0.28; 0.71	4.01E-01	5.09E-01
Lacto-N-fucopentaose V	0.28	-0.12; 0.67	1.75E-01	2.75E-01
Fucosylsialyl-lacto-N-Hexaose X2	0.36	-0.14; 0.85	1.84E-01	2.78E-01
Lacto-N-difucohexaose I	0.39	-0.12; 0.90	1.42E-01	2.34E-01
a-Heptasaccharide	0.43	-0.16; 1.03	1.61E-01	2.59E-01
Difucosyl-lacto-N-hexaose III	0.45	-0.01; 0.92	6.12E-02	1.14E-01
Lacto-N-neo-difucohexaose II	0.49	0.15; 0.84	6.07E-03	1.55E-02
a-Pentasaccharide	0.49	-0.12; 1.11	1.21E-01	2.04E-01
3-Fucosyllactose	0.50	0.24; 0.76	3.22E-04	1.23E-03
6'-Sialyl-N-acetyllactosamine	0.66	0.22; 1.09	4.31E-03	1.28E-02
Fucosyldisialyllacto-N-hexaose X2	0.68	0.35; 1.01	9.86E-05	5.00E-04
Fucosyllactose-sulfate X1	0.73	0.22; 1.24	6.04E-03	1.55E-02
6'-Galactosyllactose	0.77	0.36; 1.19	4.34E-04	1.47E-03
Disialyllacto-N-tetraose	0.87	0.52; 1.22	4.33E-06	4.39E-05
Fucosylsialyl-lacto-N-tetraose X1	0.93	0.55; 1.31	5.80E-06	4.57E-05
Fucosylsialyl-lacto-N-Hexaose X4	0.98	-2.09; 4.04	5.76E-01	6.50E-01
Lactose-3'-Sulfate	1.00	0.40; 1.61	1.68E-03	5.44E-03
Trifucosyl-lacto-N-tetraose X5	1.00	0.48; 1.53	3.47E-04	1.23E-03
2',3-Difucosyllactose	1.14	0.73; 1.56	5.91E-07	6.99E-06

Fucosylsialyl-lacto-N-tetraose a	1.24	0.91; 1.56	2.26E-11	7.76E-10
3'-Sialyl-3-fucosyllactose	1.24	0.88; 1.61	1.14E-09	1.62E-08
Fucosyldisialyl-lacto-N-tetraose X2	1.38	0.98; 1.77	5.25E-10	9.32E-09
3'-Galactosyllactose	1.59	1.16; 2.01	3.28E-11	7.76E-10
3'-sialyllactose	2.08	1.73; 2.43	4.39E-21	3.12E-19

a. Coef. refers to the coefficients of time point term in the mixed-effect models with a random intercept for each mother that accounted for maternal age during pregnancy, maternal BMI before pregnancy, parity (1 vs >1), mothers' gestational age (weeks), mothers' education level (high school or lower vs. university degree or higher), maternal smoking before pregnancy (yes vs no), delivery mode (vaginal delivery vs. cesarean delivery), and batch effect.

b. P_{FDR} refers to false discovery rate adjusted P values. P_{FDR} <0.01 is considered as the statistical significance threshold

Abbreviations: HMOs: Human milk oligosaccharides; Coef. : coefficient; CIs: confidence intervals; FDR: false discovery rate
